# High-Resolution 3D Printing Fabrication of a Microfluidic Platform for Blood Plasma Separation

**DOI:** 10.3390/polym14132537

**Published:** 2022-06-22

**Authors:** Sandra Garcia-Rey, Jacob B. Nielsen, Gregory P. Nordin, Adam T. Woolley, Lourdes Basabe-Desmonts, Fernando Benito-Lopez

**Affiliations:** 1 Microfluidics Cluster UPV/EHU, Analytical Microsystems & Materials for Lab-on-a-Chip (AMMa-LOAC) Group, Analytical Chemistry Department, University of the Basque Country UPV/EHU, 48940 Leioa, Spain; sandra.garcia@ehu.eus; 2 Microfluidics Cluster UPV/EHU, BIOMICs Microfluidics Group, Lascaray Research Center, University of the Basque Country UPV/EHU, 01006 Vitoria-Gasteiz, Spain; 3 Department of Chemistry and Biochemistry, Brigham Young University, Provo, UT 84602, USA; jbn442@gmail.com; 4 Department of Electrical and Computer Engineering, Brigham Young University, Provo, UT 84602, USA; nordin@byu.edu; 5 Bioaraba Health Research Institute, Microfluidics Cluster UPV/EHU, 01009 Vitoria-Gasteiz, Spain; 6 BCMaterials, Basque Center for Materials, Applications and Nanostructures, UPV/EHU Science Park, 48940 Leioa, Spain; 7 Basque Foundation of Science, IKERBASQUE, Calle María Díaz de Haro 3, 48013 Bilbao, Spain

**Keywords:** 3D printing, stereolithography, high resolution, fabrication, whole blood, plasma separation

## Abstract

Additive manufacturing technology is an emerging method for rapid prototyping, which enables the creation of complex geometries by one-step fabrication processes through a layer-by-layer approach. The simplified fabrication achieved with this methodology opens the way towards a more efficient industrial production, with applications in a great number of fields such as biomedical devices. In biomedicine, blood is the gold-standard biofluid for clinical analysis. However, blood cells generate analytical interferences in many test procedures; hence, it is important to separate plasma from blood cells before analytical testing of blood samples. In this research, a custom-made resin formulation combined with a high-resolution 3D printing methodology were used to achieve a methodology for the fast prototype optimization of an operative plasma separation modular device. Through an iterative process, 17 different prototypes were designed and fabricated with printing times ranging from 5 to 12 min. The final device was evaluated through colorimetric analysis, validating this fabrication approach for the qualitative assessment of plasma separation from whole blood. The 3D printing method used here demonstrates the great contribution that this microfluidic technology will bring to the plasma separation biomedical devices market.

## 1. Introduction

The growing microfluidic market demands the development of microfabrication methodologies able to improve or replace traditional fabrication technologies. In this regard, 3D printing is a candidate with great potential in the field since it offers fast production times, cost effectiveness, one-step fabrication, high resolution and minimum waste [1,2]. The fabrication flexibility and the possibility of rapid modification of designs open the path towards iterative fabrication processes. Moreover, the possibility of fabricating complex structures and the integration of functionalities inside the devices, such as valves and pumps [3,4,5], are key points that contribute to the growth of this technology. In this way, 3D printing successfully answers the increasing demand for powerful and rapid prototyping techniques for manufacturing processes.

The use of 3D printing in microfluidics has considerably increased in the last 10 years, as demonstrated by the publication trend in this field [6]. However, this technology faces some challenges that will need to be answered soon to ensure 3D printing generates a solid root in microfluidics. For example, the resolution offered by commercially available 3D printers is difficult to implement in microfluidics, which usually requires high resolution and small features [7,8].

3D printing approaches that have been successfully applied in microfluidics include inkjet 3D printing (i3DP), stereolithography (SLA), two-photon polymerization (2PP) and fused deposition modeling (FDM) [1,2]. Of these, SLA offers the best combination of resolution and 3D printing throughput [8]. In SLA, 3D structures are built in a layer-by-layer photopolymerization approach with a UV-curable liquid resin contained in a vat. In one approach, a laser sequentially scans the features of each layer, whereas in the second printing configuration, each layer is projected and polymerized simultaneously using digital light projection (DLP) [9].

Although microfluidic device components (mixers, separation chambers, sensing chambers and so on) can be used independently, their integration within a combined, multifunctional microfluidic platform makes the greatest impact. In this regard, 3D printing allows the fabrication of microfluidic devices with several integrated components, each of which can be validated independently in a fast and iterative way. In particular, the possibility of applying 3D printing to biomedicine opens new approaches for improved diagnostic analyses. However, the development of fully functional 3D-printed microfluidic platforms with integrated components is still challenging.

In particular, diagnostic tests are being successfully developed for the determination of biomarkers in blood. In this regard, 3D printing has significantly impacted this field, offering reliable and accurate biomarker determination. For instance, Tang et al. [10] developed a transparent 3D-printed microfluidic device with an integrated immunoassay to measure protein biomarkers. The same group also developed an ELISA assay in 3D-printed pipette tips for the detection of four cancer biomarker proteins [11]. Other 3D-printed approaches for blood biomarker determination include the fabrication of 3D-printed electrodes for the determination of L-methionine in biological samples [12] and the fabrication of a 3D-printed, flexible electrochemical biosensor for glucose determination [13].

However, blood cells interfere with many biomarker determinations, leading to inaccurate concentration values [14]. Therefore, plasma separation is a critical step to improve analytical performance and to develop reliable and accurate detection systems. At the microfluidic level, Tripathi et al. [15] achieved plasma separation by introducing biophysical and geometrical effects to channels, obtaining a 99% separation efficiency. In another example, Kuan et al. [16] developed a polydimethylsiloxane (PDMS) device for plasma separation based on an asymmetric bifurcation of the main channel, allowing plasma to flow through the branch with lower flow rates according to the Zweifach–Fung bifurcation law. Another successful microfluidic approach was based on the density difference of blood components, which integrated sedimentation trenches in a PDMS microfluidic device, through which the sedimentation of blood cells was demonstrated [17].

PDMS is the most commonly used fabrication material for microfluidic platforms for plasma separation. However, the use of 3D printing technology is slowly starting to gain importance in this field. Approaches such as the high-efficiency hydrophobic plasma separator developed by Liu et al. [18] using a 200 µL input of a blood sample from Schistosoma mansoni, or the 3D-printed microfluidic device for plasma separation based on centrifugal forces developed by Qin et al. [19] using a 300 µL input of a pig blood sample are a few examples found in the literature. However, the lack of testing with human whole blood hinders their applicability for biomedical testing. Moreover, the development of devices capable of analyzing lower amounts of blood, similar to those of a finger prick, would be advantageous for their future application in point-of-care devices.

In order to demonstrate the potential of 3D printing for the fabrication of plasma separation microfluidic devices, we have developed an iterative optimization process and evaluated this approach with 17 different device designs. Each device was individually tested, and the performance information was implemented in the next device configuration to generate a final operative device with multiple improved characteristics coming from previous iterations. Three different customized resin formulations were used in the research: a hydrophilic resin with a light-yellow tint (resin A), a transparent hydrophilic resin (resin B) and a transparent hydrophobic resin (resin C). The final microfluidic device was used, as a proof of concept, to separate plasma from human whole blood, in an easy and reliable way, demonstrating the potential of this stereolithographic 3D printing technology for microfabrication. This research will enable the fabrication of 3D-printed monolithic devices with integrated plasma separation components for biomarker sensing in blood.

## 2. Materials and Methods

### 2.1. Microfabrication by High-Resolution Additive Manufacturing Technology

Three different resins (resin A, resin B and resin C) were formulated for the 3D printing process, each of them composed of a monomer, a UV absorber and a photoinitiator. Resin A contained 97% poly(ethylene glycol) diacrylate (PEGDA, Sigma Aldrich, Milwaukee, WI, USA), 2% 2-nitrophenyl phenyl sulfide (NPS, 98%, TCI America, Portland, OR, USA) and 1% phenylbis(2,4,6-trimethylbenzoyl)phosphine oxide (Irg, Irgacure 819, 97%, Sigma Aldrich, Milwaukee, WI, USA). Resin B contained 98.6% PEGDA, 0.38% avobenzone (Avo, MakingCosmetics, Redmond, WA, USA) and 1% Irg. Finally, resin C contained 98.6% 1,6-hexanediol diacrylate (HDDA, Sigma Aldrich, Milwaukee, WI, USA), 0.38% Avo and 1% Irg. The chemicals for each resin were mixed and sonicated until their complete dissolution and were stored in amber bottles at room temperature (21–25 °C) until use.

Glass microscope slides (3 × 1 in, 1 mm thick, Avantor, Center Valley, PA, USA) were scored into three equal parts using a laser cutter (Universal Laser Systems, Scottsdale, AZ, USA). The scored glass slides were first washed with acetone (Fisher Scientific, Salt Lake City, UT, USA) and then with 2-propanol (IPA, Fisher Scientific, Salt Lake City, UT, USA). After drying them with compressed air, they were submerged in a 10% (*v*/*v*) solution of 3-(trimethoxy silyl)propyl methacrylate (Sigma Aldrich, Milwaukee, WI, USA) in toluene (Avantor, Center Valley, PA, USA) for 2 h. Then, the silanized glass slides were washed with IPA, air dried and broken along the scored marks. They were stored in toluene until use.

The devices were designed with an open-source CAD software (OpenSCAD, openscad.org). The microfluidic devices were fabricated with a custom-made, high-resolution DLP-SLA 3D printer, which is described in the previous literature [20,21]. The 3D printer used a 365 nm LED, a pixel size of 7.6 µm in the image plane and a layer thickness of 10 µm. The devices were built on the silanized glass, which was attached to the 3D printing platform before each print job. After printing, IPA was flushed through the channels of the devices to remove the remaining unpolymerized resin, and they were cured under a 430 nm LED (ThorLabs, Newton, NJ, USA) for 20 min.

### 2.2. Whole Human Blood

For plasma separation experiments, commercially available single-donor human whole blood with sodium citrate as an anticoagulant (Innovative Research, Novi, MI, USA) was used within 4–5 days from receipt. Whole blood and 1:1.5, 1:3, 1:4 and 1:10 dilutions in PBS (Gibco pH 7.4, 1×, Fisher Scientific, USA) were used. All solutions were kept at 2–8 °C.

### 2.3. Plasma Separation by Sedimentation

**Figure 1:**
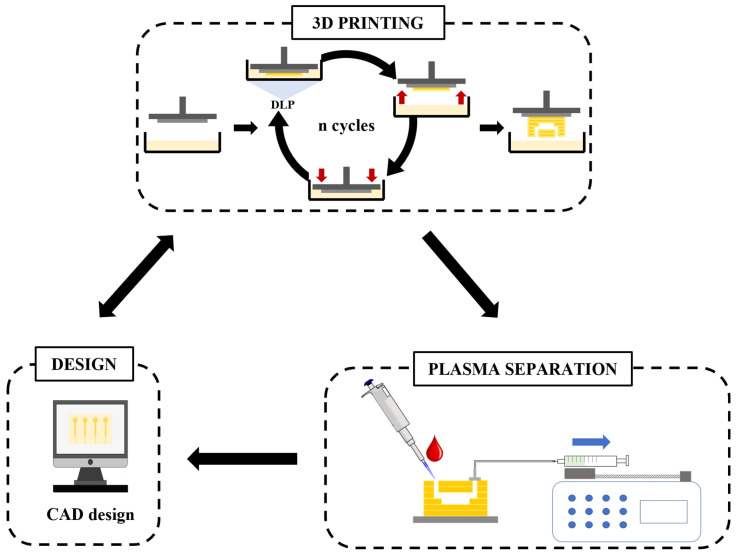
Workflow of the iterative fabrication process and the application of the fabricated microfluidic devices for plasma separation. The first step was the design of the devices through CAD software. Then, these models were 3D printed using a high-resolution, custom-made 3D printer with custom-made resins. This allowed fast fabrication times, thus the CAD designs could be easily modified based on the fabrication-and-fail optimization method, which allowed the development of an iterative fabrication process until obtaining an optimum device.

Plasma was separated from whole blood by the sedimentation of the red blood cells (RBCs) in the trenches of the microfluidic devices. Before using, the devices were washed three times with IPA and twice with PBS. A syringe pump (Chemyx, Fusion Touch, version 1.7.6 c) was used to control the flow, using either positive or negative pressure, in the 1–15 µL min^−1^ range at a constant rate. In addition, 250 µL syringes (Hamilton, Reno, NV, USA) were used to introduce the sample into the devices. PTFE tubing (0.22 in ID × 0.042 in OD, Cole-Parmer, Vernon Hills, IL, USA) and capillary tubing (polymicro flexible fused silica capillary tubing, ID 50 µm, OD 375 µm, Lisle, IL, USA) were fixed with UV-curable resin (DecorRom, Shenzhen, China) to connect the microfluidic devices to the syringe pump. The entire process is summarized in Figure 1.

### 2.4. Image Analysis

Images were taken during experiments, using a 20 MP (megapixel) + 2 MP dual camera with f/1.8 aperture (Huawei). The images were analyzed using the color analysis software ImageJ. The original color image was converted first to an 8-bit grayscale image and, then, the black and white (B/W) value was measured, scaling from 0–255, where 0 stands for black and 255 for white. Both the whole blood input sample and the separated plasma were measured, allowing the determination of the quality of the plasma through image analysis.

## 3. Results and Discussion

### 3.1. Iterative 3D Printing Fabrication Process

A total of 17 different device designs were fabricated by additive manufacturing technology with printing times ranging from 5–12 min. These devices were originally designed to be independent microfluidic modules to separate plasma from a drop-like input blood volume, which could then be coupled into a larger microfluidic device in which a plasma separation step is required. Table 1 summarizes the principal characteristics of the prototypes; for more detailed information, see the Supplementary Information Tables SI-1–6. All the devices were designed and fabricated in an iterative process, using a testing-based optimization strategy, demonstrating the great potential of this novel technology in the microfluidic field as an easy and reliable fabrication method.

In order to explore the use of 3D printing materials and their effect on plasma separation, three different resin formulations were employed for device fabrication. Devices 1–15 were printed with a hydrophilic resin containing NPS (resin A, see Section 2.1), which gave them a yellowish appearance. This effect was already observed in previous research, which used the same resin formulation [22,23]. For Device 16, NPS was replaced by Avo (resin B) which, unlike resin A, gave the devices a transparent appearance, facilitating the subsequent colorimetric analysis of the separated plasma when performed in situ in the device. For Device 17, the monomer was changed to HDDA (resin C), which gave the device hydrophobic properties.

Although not all the formulations worked for the purpose of this research (see SI-7), the results demonstrated the potential of customizing the device materials using resins with application-targeted properties. The use of custom-made resins represents an advantage over commercially available ones since it allows the user to modulate the properties of the microfluidic device, such as its transparency or physicochemical surface characteristics [8]. In this field, novel materials are already being developed, such as the biocompatible PEGDA resin developed by Warr et al. [24] and the gelatin methacryloyl-based bioink developed by Kumar et al. [25].

### 3.2. Round-Like Sedimentation Trench (Devices 1–3)

**Figure 2:**
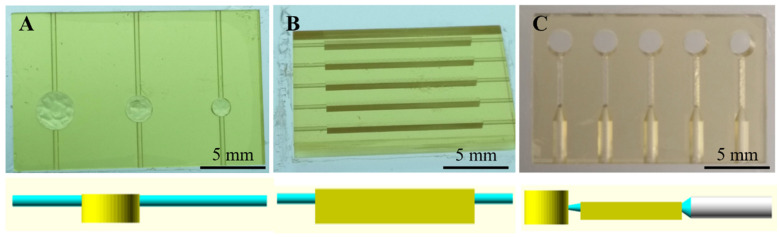
3D-printed devices (resin A) and the CAD design of the lateral view of the channels. Device 3 (**A**) consisted of round-like chambers and three independent channels. Devices 4 (**B**) and 14 (**C**) consisted of rectangle-like sedimentation trenches and five independent channels.

To test for a channel and trench geometry that provided the best plasma separation, as well as an appropriate liquid flow, the first designs (Devices 1–3) were fabricated with a round trench configuration. Each device had three channels, which could be operated independently, and three reservoirs with diameters ranging from 0.74 mm to 1.47 mm. Figure 2A shows an image of Device 3 and the schematic diagram of the side view of the channels. For more information about Devices 1 and 2, see Table SI-1.

As can be appreciated in Figure 2A, the top layer of the sedimentation chambers was partially delaminated in the case of the round-like chambers, whereas the bottom layers remained smooth. The same phenomenon was observed in Devices 1 and 2. At first, it was thought that this heterogeneity in the layers was due to the flushing out of the remaining resin with 2-propanol after the printing process. Due to the small dimensions of the channels in the first two devices, it was difficult to remove unpolymerized resin from the round-like trenches, giving rise to the polymerization of residual resin in the chambers during the post-curing step. For this reason, the channel dimensions were redesigned to be larger in Devices 2 and 3 to improve flushing of the resin out of the device chamber and channels. However, the same delamination was observed even in Device 3, except for with the smallest chamber. Contrary to what it was initially thought, delamination did not occur during the cleaning step of the printed device, but during the printing process itself. Since the devices were printed upside-down, the bottom layers of the device were the first ones to be printed and were attached to a solid base. The top layers, however, were the last ones to be printed and did not have a solid base to be printed on. Consequently, they tended to delaminate into the channel and interfere with the flow. This acted as a limitation for the microfabrication of the devices, setting the maximum diameter of the chamber that could successfully be printed to be 1.04 mm.

### 3.3. Rectangle-Like Sedimentation Trench (Devices 4–9)

To reduce the delamination of the top layers of the sedimentation chambers, subsequent devices were designed with rectangle-shaped trenches. The first prototype of this generation was Device 4, which is shown in Figure 2B. This device consisted of five independent channels with identical sedimentation trenches, which were 13.68 mm long by 0.40 mm wide by 1.40 mm tall. Maintaining the same trench dimensions, Device 5 was designed to have two separated channels with two and three serially connected trenches, respectively. Additionally, Device 6 had one channel with the five serially connected trenches as a single device (see Table SI-2).

Devices 4 and 5 were tested with diluted blood samples by applying positive pressure. This configuration, however, did not yield optimal plasma separation in the device because of initial sedimentation in the tubing that started before the sedimentation trench, as explained later in Section 3.6. Consequently, the following devices were designed to be operated by applying negative pressure.

Moreover, based on these results, it was concluded that devices with more than two sedimentation chambers were not necessary. Connected sedimentation chambers require larger sample volumes, which may limit work with real samples that contain very low concentrations of target analyte. For example, Almughamsi et al. [26] used 12 µL of human blood serum with three different antibodies (1 mg mL^−1^) as the input sample for a multiplexed extraction of preterm birth biomarkers in 3D-printed microfluidic devices. Therefore, Device 6 was not tested for sedimentation in this study since it was designed to accommodate larger input samples. Although these types of configurations were not suitable for this particular research, devices with similar features could be applied in other microfluidic systems in which larger blood volumes are required [27].

Devices 7–9 (see Table SI-3), were designed to have smaller sedimentation trenches, but in higher numbers, to improve plasma separation. However, the addition of more than one sedimentation chamber added difficulty for blood flow through the device and, thus, the final plasma separation. This device configuration was considered to be more appropriate for larger blood samples, in which blood cells would sediment into the bottom of the small trenches, become trapped and not flow into the next sedimentation trenches. This approach would increase the efficiency of separation by allowing clearer plasma, as the blood flowed through the different trenches, until obtaining fully separated plasma. Therefore, Devices 7–9 were printed but not tested here.

### 3.4. Towards the Final Prototype (Devices 10–14)

Once the separation chamber was successfully developed, Devices 10–12 were designed to investigate the connection between the inlet reservoir and the sedimentation trench (see Table SI-4), which had a vertical zig-zag shape. During testing of these devices with whole blood, plasma could not be separated since the blood became stuck in the connecting channel. These results demonstrated that the best configuration consisted of a straight channel connecting the inlet reservoir with the sedimentation trench, a configuration that was implemented in the next devices. This configuration allowed the sedimentation of the RBC process to begin in the inlet reservoir, as later explained in Section 3.6, and the length of the sedimentation trenches could be reduced.

The connectivity of the device with the pumping system was also investigated at this point. For Devices 13 and 14 (Table SI-5), which were operated by negative pressure and connected to a syringe pump using PTFE tubing, the configuration did not work well with the collecting channel of the devices, resulting in many leaks during operation. Considering that fact, the outlet connection of the next devices was designed to fit capillary tubing instead the PTFE one, which could be better affixed to the device. Device 14 is shown in Figure 2C.

### 3.5. Optimized Microfluidic Device for Plasma Separation (Device 15)

**Figure 3:**
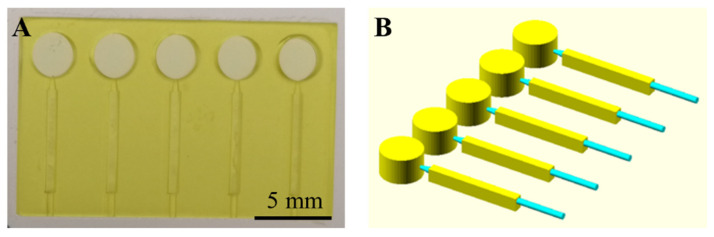
Optimized 3D-printed microfluidic prototype (Device 15) for plasma separation. (**A**) Top view image of the final device. (**B**) CAD design of the separated channels.

The optimized design (Device 15) is shown in Figure 3, with more details in Table SI-5. The microfluidic device (19.45 × 12.16 × 1.80 mm) contained five separated channels, each of which consisted of an inlet reservoir capable of holding 12 µL of sample, which was connected to the sedimentation trench (6.27 × 0.61 × 0.90 mm). In this optimized prototype, the sedimentation trenches had a rectangular configuration, and the channels were connected through capillary tubing and operated by negative pressure.

### 3.6. Plasma Separation

Plasma separation was achieved by the sedimentation of the RBCs to the bottom of the sedimentation trench due to their higher density [17,25]. Table 2 shows the performance specifications for Device 3 (see Section 3.1), Device 4 (see Section 3.2), Device 14 (see Section 3.3) and the optimized Device 15 for plasma separation (see Section 3.4) as examples.

In Devices 1–9, the sample was loaded in a syringe, which was then connected to the microfluidic device through PTFE tubing. Then, positive pressure was used to flow the sample into the device. Although plasma could be separated in Device 4 by using this setup, the separation was not optimal since it could only be achieved when using diluted blood samples. Moreover, when using positive pressure to drive the flow, plasma also began to separate in the syringe even before reaching the sedimentation trench. Consequently, it could not be verified if the plasma obtained from these devices was, in fact, due to separation inside the device. Therefore, the next designs (Devices 10–17) were operated with negative pressure. Device 14 was tested with this configuration, but PTFE tubing was used as the outlet connection to the pumping system (see Section 3.4), which led to leaks. Consequently, the outlet connection was replaced by capillary tubing in Device 15, yielding a successful microfluidic device operation for plasma separation.

**Figure 4:**
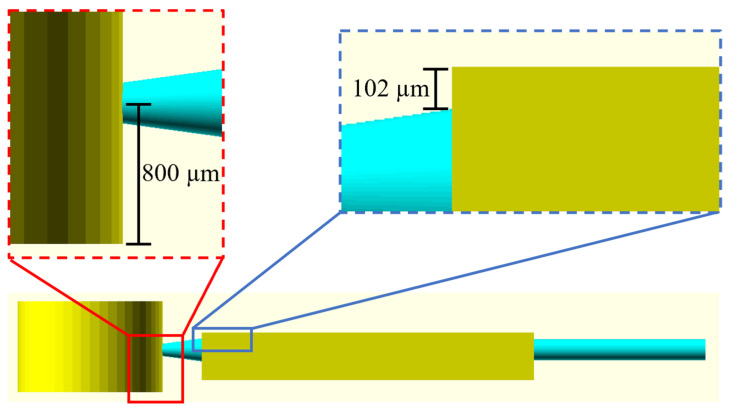
CAD design of Device 15 with the connecting channel between the inlet reservoir and the sedimentation trench placed 800 µm above the bottom layer of the reservoir (red), and a hydrophobic air barrier of 102 µm placed at the top of the sedimentation trench (blue) to enhance separation of plasma from whole blood.

In Device 15, to avoid having the blood cells stick to the top of the trench, it was necessary to introduce a hydrophobic barrier (Figure 4), as previously demonstrated by Dimov et al. [17]. To achieve this, the top layer of the trench was located ~100 µm above the entrance of the liquid into the chamber and, thus, a hydrophobic air barrier was generated. Moreover, to enhance the separation of plasma, the channel connecting the inlet reservoir and the sedimentation trench was placed 800 µm above the bottom of the inlet reservoir (Figure 4). This allowed the inlet reservoir to act as a sedimentation trench as well, where the RBCs that sedimented there during device operation did not enter the sedimentation chamber at all, improving the quality of the separated plasma.

**Figure 5:**
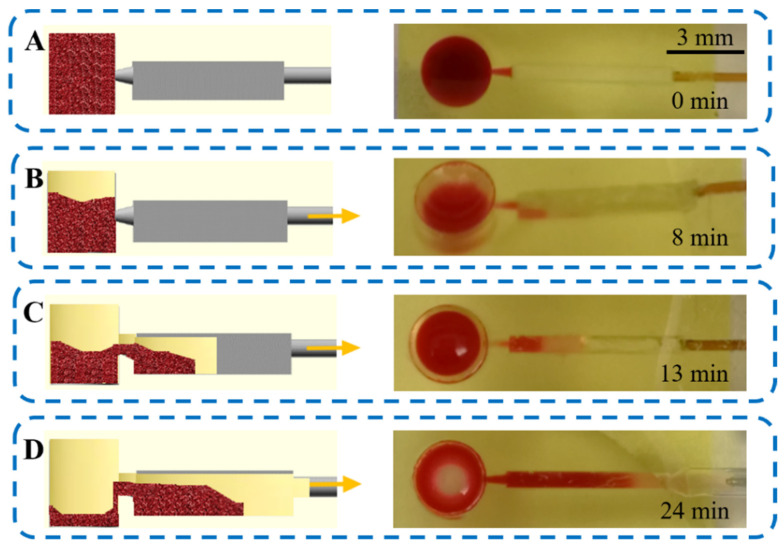
Workflow for plasma separation, showing a schematic diagram (side view) and top-view photos of the 3D-printed Device 15 during each step of the process. (**A**) First, 12 µL of whole human blood was loaded in the input reservoir and let stand for 8 min. (**B**) Then, negative pressure (yellow arrow) was applied at a constant flow of 1 µL min^−1^ and the sample entered the sedimentation trench. (**C**) Blood cells sedimented to the bottom whereas plasma was separated due to its lower density. (**D**) Finally, the plasma was collected from the outlet to be further analyzed.

Figure 5 shows the performance of Device 15 during the separation of plasma from whole blood. The followed approach was based on a previous work by Dimov et al. [17], where they achieved plasma separation through sedimentation and biomarker detection in an integrated microfluidic platform. In the sedimentation trench, plasma can be separated from whole blood under the influence of gravity, where RBCs (together with white blood cells) sediment to the bottom of the trench due to their higher density as compared to the other components of blood. This sedimentation process is classified as a passive technique for plasma separation since it does not require any external input or centrifugation. In our research, we have integrated this effect in a microfluidic platform, where sedimentation occurs both in the inlet and, at a major scale, in the sedimentation trench.

Therefore, after loading 12 µL of whole human blood in Device 15, a waiting time of 8 min was set before starting the flow to allow initial sedimentation of the RBCs in the inlet reservoir. The microfluidic device was operated at a constant flow rate of 1 µL min^−1^ for 35 min by applying negative pressure. Blood cells sedimented to the bottom, whereas plasma did not due to its lower density, instead flowing through the medium-upper section of the trench. The sedimentation chamber was filled in 16 min, and the separation of plasma could be observed even during the first 5 min of operation. Finally, the plasma was collected from the outlet to be analyzed outside the device to remove interference from the color of the resin, which hindered the visualization and colorimetric analysis of the separated plasma in the device. A volume of ~5 µL of plasma was collected. Optically transparent resins were also studied (see SI-7) as a possibility to allow the *in situ* analysis of the separated plasma.

For experiments that require longer performance times, the evaporation of the sample in the inlet could be a problem for the operation of the device. However, we did not observe any significant evaporation, demonstrated by the lack of clogging in the connecting channel, between the inlet and the sedimentation chamber, or stopping of the flow of plasma separation. Nevertheless, for future experiments which require higher input sample volumes or longer performance times, it would be advisable to consider this phenomenon.

**Figure 6:**
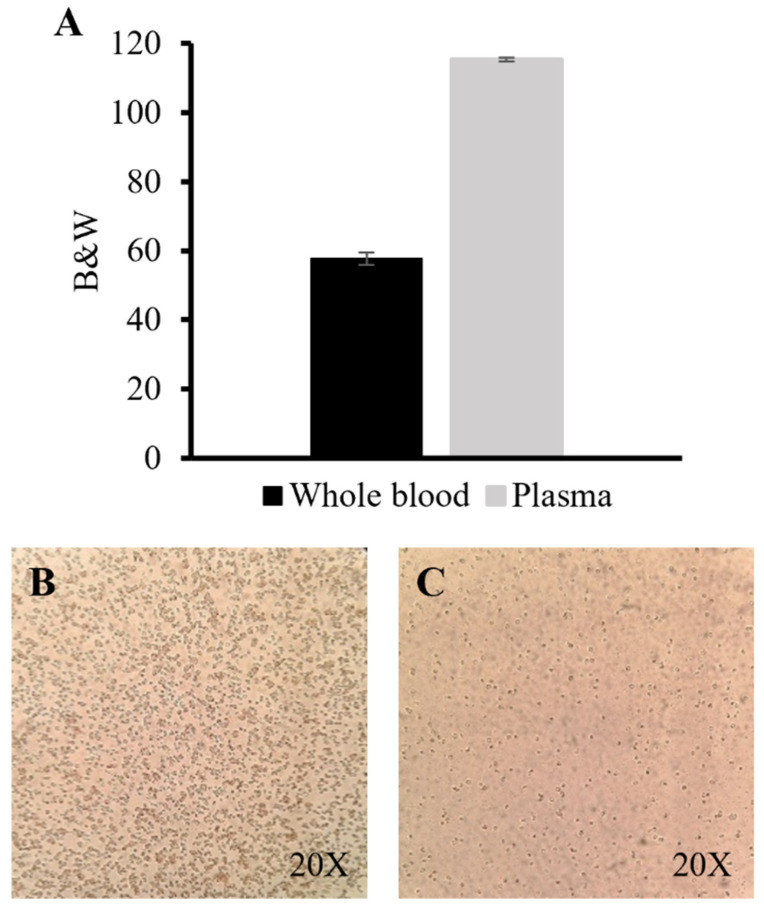
Qualitative assessment of the separated plasma through image analysis. (**A**) Black and white (B&W) analysis of whole blood (dark gray) and the separated plasma (light gray), where 0 = black and 255 = white (*n* = 3). Images at 20× magnification of a whole blood (**B**) and a separated plasma (**C**) sample drop on a slide.

A qualitative assessment of the performance of the optimized 3D-printed device (Device 15) was carried out by the colorimetric analysis of the separated plasma. As shown in Figure 6A, the B&W values obtained for the whole blood and the separated plasma were 58 ± 2 and 115 ± 1, respectively (*n* = 3) (see Section 2.4 to understand how those values were obtained). The B&W values can be related to the concentration of RBCs presented in the plasma sample, where samples with a lower number of RBCs obtained higher B&W values. Figure 6B,C show images of recovered plasma samples before and after the separation process in Device 15, respectively. RBCs can be observed as small black dots in both images. Our results demonstrated that after plasma separation, the B&W values of the obtained plasma doubled in comparison to those obtained with the whole blood samples. Therefore, high B&W values can be related to a lower number of RBCs in the sample, as seen in Figure 6C, due to the separation process. This assay demonstrates the capacity of this colorimetric image analysis method to be used as a qualitative tool to measure plasma separation. Nevertheless, the performance of these types of microfluidic devices is still far from obtaining the same quality of plasma than traditional centrifugation methods, where standardized methods are able to obtain negligible numbers of RBCs in the separated plasma.

At present, plasma is mainly separated through centrifugation for analytical determinations. However, this method needs batch processing, large volume samples and specific equipment with trained personnel [14]. As an alternative, we have demonstrated that our microfluidic device can perform plasma separation efficiently. Moreover, the small input sample needed to obtain the plasma will facilitate its analysis at the time and place needed, offering point-of-need solutions and reliable biomarker detection.

The optimized device was tested as an independent microfluidic system configuration for plasma separation. However, it could also be used as a modular platform to allow the integration of plasma separation in a larger microfluidic system, which could be applied for analytical purposes. For example, this device could be integrated with previous methodologies developed in our group for the assessment of preterm birth risk [22,23,26]. The unification of all the functionalities in the same analytical microfluidic platform will decrease the variability of biomarker measurements and would allow its integration within a single flow process, thus creating robust and reliable solutions for faster diagnosis in biomedicine.

## 4. Conclusions

Three-dimensional printing has become a powerful alternative method for microfabrication. The possibility of realizing fast modifications of the CAD designs has reinforced the success of this methodology. In this regard, an iterative fabrication process of evaluating 17 different devices, from design to performance verification, based on an optimization approach using a high-resolution, custom-made SLA 3D printer was carried out. The optimized device was then successfully applied for plasma separation from whole human blood, validating the device for the qualitative assessment of plasma separation. Using a colorimetric analysis approach, we obtained that the amount of RBCs in the separated plasma was reduced to half their value in the input sample. With only 12 µL of input sample required, this microfluidic device module could be applied at the point of need, with minimum invasiveness of the sample.

It is anticipated that this 3D printing technology will make a significant impact in microfabrication, allowing the accurate generation of features as small as 10 µm. Moreover, the fast printing times that can be achieved compared to traditional microfabrication techniques establishes 3D printing as a fabrication methodology with great potential in the microfluidic field [28,29,30]. Although it is not yet a high-throughput microfabrication technology since it is limited to the fabrication of a few devices at once, 3D printing is a great candidate to lead the way towards prototype development and manufacturing, a feature that will boost the microfluidic field to a new level.

## Figures and Tables

**Table 1:** Fabrication specifications of the 3D-printed devices.

Design	Working Pressure	Resin Composition	Resin Character	Trench Shape	Plasma Separation
1	Positive	PEGDA, NPS, Irg	Hydrophilic	Circular	N/A
2	Positive	PEGDA, NPS, Irg	Hydrophilic	Circular	N/A
3	Positive	PEGDA, NPS, Irg	Hydrophilic	Circular	N/A
4	Positive	PEGDA, NPS, Irg	Hydrophilic	Rectangle	Bad
5	Positive	PEGDA, NPS, Irg	Hydrophilic	Rectangle	Bad
6	Positive	PEGDA, NPS, Irg	Hydrophilic	Rectangle	N/A
7	Positive	PEGDA, NPS, Irg	Hydrophilic	Rectangle	N/A
8	Positive	PEGDA, NPS, Irg	Hydrophilic	Rectangle	N/A
9	Positive	PEGDA, NPS, Irg	Hydrophilic	Rectangle	N/A
10	Negative	PEGDA, NPS, Irg	Hydrophilic	Rectangle	N/A
11	Negative	PEGDA, NPS, Irg	Hydrophilic	Rectangle	N/A
12	Negative	PEGDA, NPS, Irg	Hydrophilic	Rectangle	N/A
13	Negative	PEGDA, NPS, Irg	Hydrophilic	Rectangle	N/A
14	Negative	PEGDA, NPS, Irg	Hydrophilic	Rectangle	N/A
15	Negative	PEGDA, NPS, Irg	Hydrophilic	Rectangle	Good
16	Negative	PEGDA, Avo, Irg	Hydrophilic	Rectangle	N/A
17	Negative	HDDA, Avo, Irg	Hydrophobic	Rectangle	N/A

PEGDA: poly(ethylene glycol) diacrylate, NPS: 2-nitrophenyl phenyl sulfide, Irg: phenylbis(2,4,6-trimethylbenzoyl) phosphine oxide, Avo: avobenzone, HDDA: 1,6-hexanediol diacrylate, N/A: not achieved.

**Table 2:** Performance specifications of Devices 3, 4, 14 and 15.

	Device 3	Device 4	Device 14	Device 15
Inlet reservoir volume	N/A	N/A	12 µL	12 µL
Sedimentation trench volume/µL	1.72–6.79	7.66	3.34	5.20
Sample	Distilled water	Diluted human blood	Whole human blood	Whole human blood
Working pressure	Positive	Positive	Negative	Negative
Flow rate/µL min^−1^	15	2	2	1
Plasma separation	No	Yes	No	Yes

N/A: not applicable.

## Supplementary Materials

The following supporting information can be downloaded at: https://www.mdpi.com/article/10.3390/polym14132537/s1. Table SI-1: Devices 1–3: design and specifications of the 3D printed devices; Table SI-2: Devices 4–6: design and specifications of the 3D printed devices; Table SI-3: Devices 7–9: design and specifications of the 3D printed devices; Table SI-4: Devices 10–12: design and specifications of the 3D printed devices; Table SI-5: Devices 13–15: design and specifications of the 3D printed devices; Table SI-6: Devices 16–17: design and specifications of the 3D printed devices; Section SI-7: Other microfluidic devices for plasma separation.
