# Supplementary material for: High-Resolution 3D Printing Fabrication of a Microfluidic Platform for Blood Plasma Separation
Source: Polymers (Basel). 2022 Jun 22;14(13):2537. doi: 10.3390/polym14132537 (PMC9269563; doi:10.3390/polym14132537)
Supplement: Supplementary file 1 [file polymers-14-02537-s001.zip › polymers-1723887-supplementary.pdf]

High-resolution 3D printing fabrication of a microfluidic platform for plasma separation

Sandra Garcia-Rey[a,b], Jacob B. Nielsen[c], Gregory P. Nordin[d], Adam T. Woolley[c,*], Lourdes Basabe-Desmonts[b,e, f, g,*], Fernando Benito-Lopez,[a,e,f,*]

[a]Microfluidics Cluster UPV/EHU, Analytical Microsystems & Materials for Lab-on-a-Chip (AMMa-LOAC) Group, Analytical Chemistry Department, University of the Basque Country UPV/EHU, Spain

[b]Microfluidics Cluster UPV/EHU, BIOMICs microfluidics Group, Lascaray Research Center, University of the Basque Country UPV/EHU, Vitoria-Gasteiz, Spain

[c]Department of Chemistry and Biochemistry, Brigham Young University, Provo, UT 84602, USA

[d]Department of Electrical and Computer Engineering, Brigham Young University, Provo, UT 84602, USA

[e]Bioaraba Health Research Institute, Microfluidics Cluster UPV/EHU, Vitoria-Gasteiz, Spain

[f]BCMaterials, Basque Center for Materials, Applications and Nanostructures, UPV/EHU Science Park, Leioa, Spain

[g]Basque Foundation of Science, IKERBASQUE, Calle María Díaz de Haro 3, 48013 Bilbao, Spain

Supplementary Information.

Table SI-1. Devices 1 – 3: design and specifications of the 3D printed devices.

	Design 1	Design 2	Design 3
**Designs**	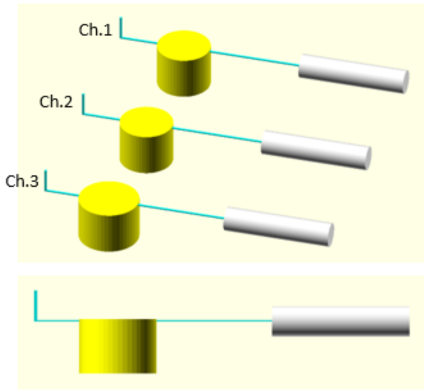	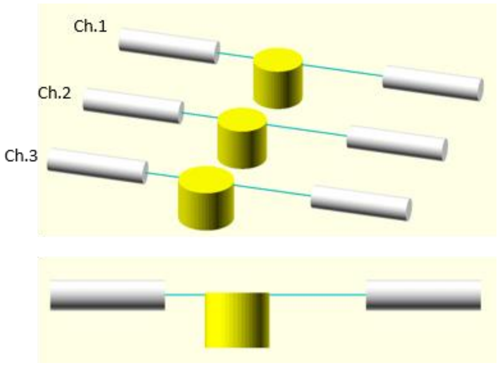	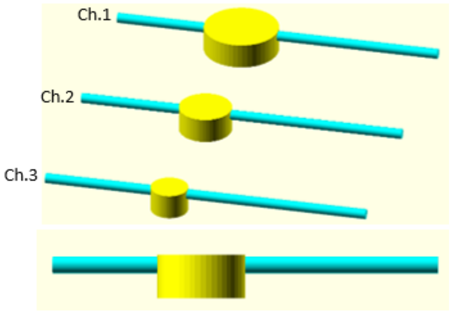
**Dimensions**	19.45 mm x 12.0 mm x 3.0 mm	19.45 mm x 12.0 mm x 3.0 mm	19.45 mm x 12.0 mm x 1.25 mm
**Channel**	90 mm x 0.05 mm	90 mm x 0.05 mm	d = 0.4 mm
**Sedimentation trench**	(Ch. 1,2) d = 1.23 mm, h = 2 mm<br>(Ch. 3) d = 1.40 mm, h = 2 mm	(Ch. 1,2) d = 1.23 mm, h = 2 mm<br>(Ch. 3) d = 1.40 mm, h = 2 mm	(ch. 1) d = 1.47 mm, h = 1 mm<br>(ch. 2) d = 1.04 mm, h = 1 mm<br>(ch. 3) d = 0.74 mm, h = 1 mm
**Printing time**	10 min	10 min	6 min
**Resin formulation**	97% PEGDA + 2% NPS + 1% Irgacure819	97% PEGDA + 2% NPS + 1% Irgacure819	97% PEGDA + 2% NPS + 1% Irgacure819

Table SI-2. Devices 4 – 6: design and specifications of the 3D printed devices.

	Design 4	Design 5	Design 6
**Designs**	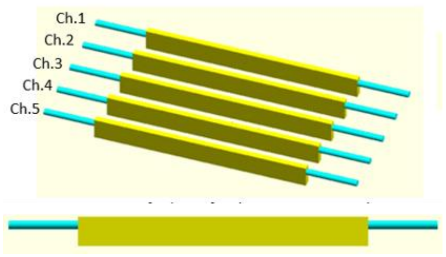	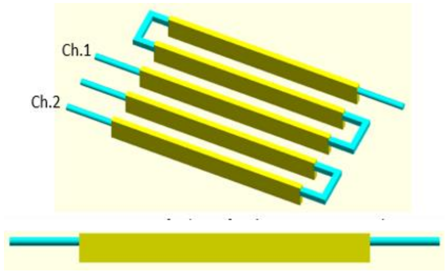	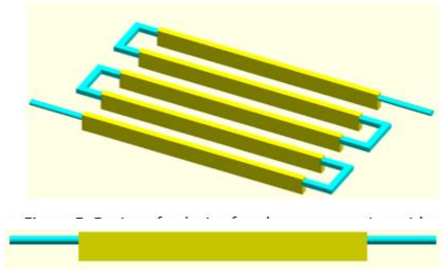
**Dimensions**	19.45 mm x 12.0 mm x 1.8 mm	19.45 mm x 12.0 mm x 1.8 mm	19.45 mm x 12.0 mm x 1.8 mm
**Channel**	d = 0.4 mm	d = 0.4 mm	d = 0.4 mm
**Sedimentation trench**	Five separated channels<br>l = 13.68 mm, w = 0.4 mm, h = 1,4 mm	Two channels with 3 and 2 connected trenches, respectively<br>l = 13.68 mm, w = 0.4 mm, h = 1,4 mm	Five connected trenches<br>l = 13.68 mm, w = 0.4 mm, h = 1,4 mm
**Printing time**	7 min	7 min	6 min
**Resin formulation**	97% PEGDA + 2% NPS + 1% Irgacure819	97% PEGDA + 2% NPS + 1% Irgacure819	97% PEGDA + 2% NPS + 1% Irgacure819

Table SI-3. Devices 7 – 9: design and specifications of the 3D printed devices.

	Design 7	Design 8	Design 9
**Designs**	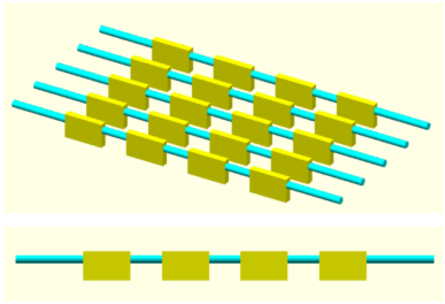	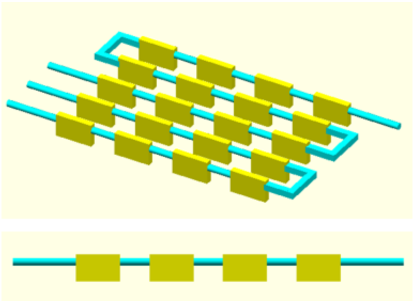	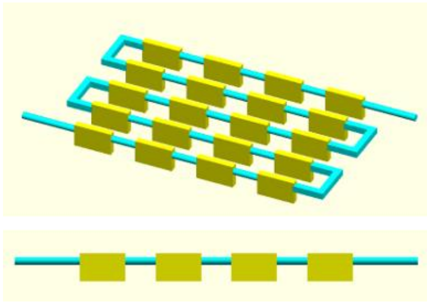
**Dimensions**	19.45 mm x 12.0 mm x 1.8 mm	19.45 mm x 12.0 mm x 1.8 mm	19.45 mm x 12.0 mm x 1.8 mm
**Channel**	d = 0.4 mm	d = 0.4 mm	d = 0.4 mm
**Sedimentation trench**	Five separated channels. Multitrench<br>l = 13.68 mm, w = 0.4 mm, h = 1,4	Two channels with 3 and 2 connected trenches, respectively. Multitrench.<br>l = 13.68 mm, w = 0.4 mm, h = 1,4	Five connected trenches. Multitrench<br>l = 13.68 mm, w = 0.4 mm, h = 1,4 mm
**Printing time**	7 min	6 min	7 min
**Resin formulation**	97% PEGDA + 2% NPS + 1% Irgacure819	97% PEGDA + 2% NPS + 1% Irgacure819	97% PEGDA + 2% NPS + 1% Irgacure819

Table SI-4. Devices 10 – 12: design and specifications of the 3D printed devices.

	Design 10	Design 11	Design 12
**Designs**	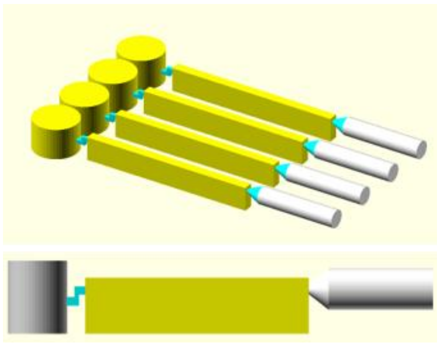	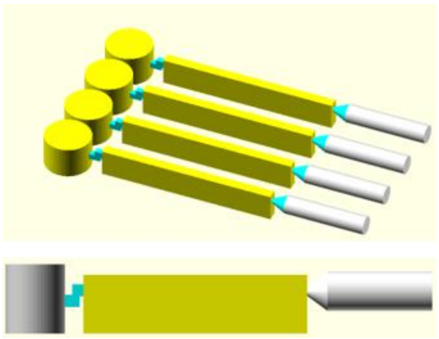	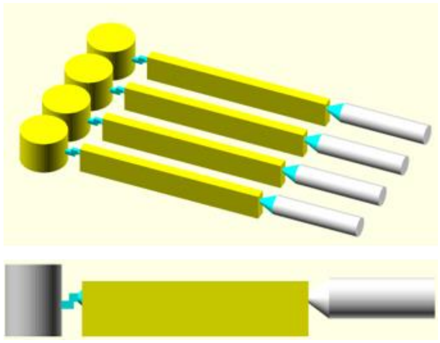
**Dimensions**	19.45 mm x 12.0 mm x 2.0 mm	19.45 mm x 12.0 mm x 2.0 mm	19.45 mm x 12.0 mm x 2.0 mm
**Channel**	0.225 mm x 0.230 mm	0.375 mm x 0.345 mm	0.375 mm x 0.345 mm, truncated cylinder connection
**Sedimentation trench**	l = 10.54 mm, w = 0.60 mm, h = 1.50 mm	l = 10.54 mm, w = 0.60 mm, h = 1.50 mm	l = 10.54 mm, w = 0.60 mm, h = 1.50 mm
**Printing time**	9 min	9 min	9 min
**Resin formulation**	97% PEGDA + 2% NPS + 1% Irgacure819	97% PEGDA + 2% NPS + 1% Irgacure819	97% PEGDA + 2% NPS + 1% Irgacure819

Table SI-5. Devices 13 – 15: design and specifications of the 3D printed devices.

Designs	Design 13	Design 14	Design 15
	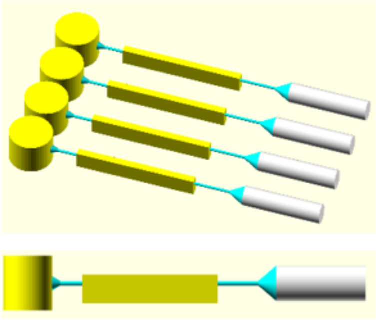	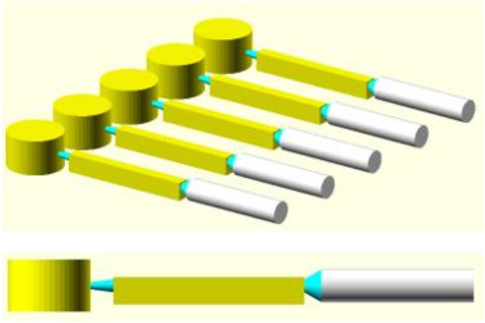	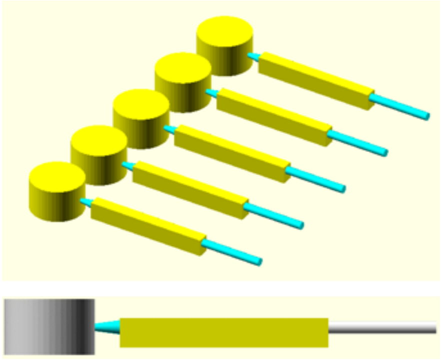
**Dimensions**	19.45 mm x 12.0 mm x 1.8 mm	19.45 mm x 12.0 mm x 1.8 mm	19.45 mm x 12.0 mm x 1.8 mm
**Channel**	d = 0.15 mm	d = 0.15 mm	d = 0.15 mm
**Sedimentation trench**	l = 6.18 mm, w = 0.60 mm, h = 0.90 mm	l = 6.18 mm, w = 0.60 mm, h = 0.90 mm	l = 7.25 mm, w = 0.60 mm, h = 1.20 mm
**Printing time**	8 min	8 min	8 min
**Resin formulation**	97% PEGDA + 2% NPS + 1% Irgacure819	98.6% PEGDA + 0.38% Avo + 1% Irgacure819	97% PEGDA + 2% NPS + 1% Irgacure819

Table SI-6. Devices 16 – 17: design and specifications of the 3D printed devices.

**Designs**	**Design 16**	**Design 17**
	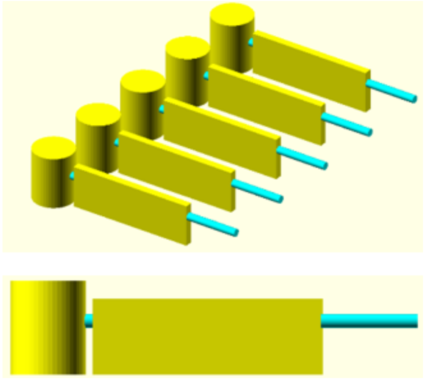	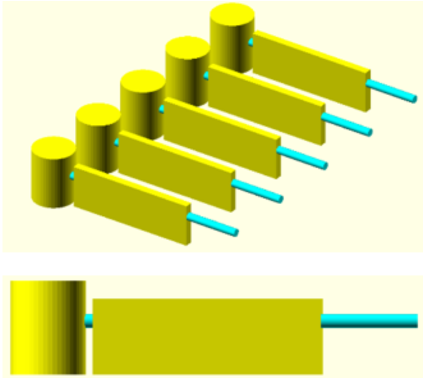
**Dimensions**	19.45 mm x 12.0 mm x 2.8 mm	19.45 mm x 12.0 mm x 1.8 mm
**Channel**	d = 0.4 mm	d = 0.4 mm
**Sedimentation trench**	l = 6.58 mm, w = 0.4 mm, h = 2.20 mm	l = 6.84 mm, w = 5.7 mm, h = 1.3 mm
**Printing time**	11 min	9 min
**Resin formulation**	98.6% PEGDA + 0.38% Avo + 1% Irgacure819	98.6% HDDA + 0.38% Avo + 1% Irgacure819

SI-7. Other microfluidic devices for plasma separation

In an effort to improve the separation process, two additional resin formulations were investigated. These, two alternative microfluidic devices were tested, which had different material properties from Device 15, but with a similar structure (see Table SI-6). The first approach involved the fabrication of a transparent device to overcome the need for the collection of the separated plasma for colorimetric analysis in Device 15 (see Section 3.6). A transparent device should allow *in situ* colorimetric analysis of the separated plasma in the device. For this purpose, Device 16 was successfully fabricated, see Table SI-6, with resin B. However, during the evaluation of this device, whole blood did not enter the sedimentation trench and plasma separation was not achieved. A possible explanation for this fact could be the chemical difference between the UV absorbers, Avo and NPS, which could give the resin different surface chemistry properties. Another possibility is that the change in UV absorber resulted in small variations in 3D printed feature dimensions or surface roughness, which could also affect flow.

For Device 17, a hydrophobic resin (resin C) was developed in the pursuit of mimicking the hydrophobic character of the barrier used in Device 15. Because this feature was important for successful sedimentation, we fabricated a hydrophobic device to test this feature for the entire device. However, we found that the blood sample did not flow into Device 17. Therefore, we concluded that, the same feature that allowed the blood cells not to stick to the top surface of the sedimentation trench and thus, a successful plasma separation, acted as an obstacle for the entrance of the whole blood to this chamber. The highly hydrophobic character of Device 17 could be the reason behind that, which hindered the blood flow, making impossible the separation of the plasma.
